# Apolipoprotein E - A Multifunctional Protein with Implications in Various Pathologies as a Result of Its Structural Features

**DOI:** 10.1016/j.csbj.2017.05.003

**Published:** 2017-06-06

**Authors:** Irina Florina Tudorache, Violeta Georgeta Trusca, Anca Violeta Gafencu

**Affiliations:** Institute of Cellular Biology and Pathology “Nicolae Simionescu” of the Romanian Academy, 8 B. P. Hasdeu Street, Sector 5, 050568 Bucharest, Romania

**Keywords:** ApoE, Apolipoprotein E, AD, Alzheimer's disease, CVD, cardiovascular disease, HCV, hepatitis C virus, HSPGs, heparan sulfate proteoglycans, HDL, high-density lipoprotein, HIV, human immunodeficiency virus, HSV-1, herpes simplex virus type-1, LPG, lipoprotein glomerulopathy, LPL, lipoprotein lipase, LDL, low density lipoprotein, NS5A, nonstructural protein 5A, HLP, phospholipid transfer protein, PLTP, type III hyperlipoproteinemia, TG, triglyceride, VLDL, very-low-density lipoprotein, ApoE, Isoform, Mimetic peptide, Structural domain, Truncated molecule

## Abstract

Apolipoprotein E (apoE), a 34 kDa glycoprotein, mediates hepatic and extrahepatic uptake of plasma lipoproteins and cholesterol efflux from lipid-laden macrophages. In humans, three structural different apoE isoforms occur, with subsequent functional changes and pathological consequences. Here, we review data supporting the involvement of apoE structural domains and isoforms in normal and altered lipid metabolism, cardiovascular and neurodegenerative diseases, as well as stress-related pathological states. Studies using truncated apoE forms provided valuable information regarding the regions and residues responsible for its properties. ApoE3 renders protection against cardiovascular diseases by maintaining lipid homeostasis, while apoE2 is associated with dysbetalipoproteinemia. ApoE4 is a recognized risk factor for Alzheimer's disease, although the exact mechanism of the disease initiation and progression is not entirely elucidated. ApoE is also implicated in infections with herpes simplex type-1, hepatitis C and human immunodeficiency viruses. Interacting with both viral and host molecules, apoE isoforms differently interfere with the viral life cycle. ApoE exerts anti-inflammatory effects, switching macrophage phenotype from the proinflammatory M1 to the anti-inflammatory M2, suppressing CD4+ and CD8+ lymphocytes, and reducing IL-2 production. The anti-oxidative properties of apoE are isoform-dependent, modulating the levels of various molecules (Nrf2 target genes, metallothioneins, paraoxonase). Mimetic peptides were designed to exploit apoE beneficial properties. The “structure correctors” which convert apoE4 into apoE3-like molecules have pharmacological potential. Despite no successful strategy is yet available for apoE-related disorders, several promising candidates deserve further improvement and exploitation.

## 1 Introduction

Apolipoprotein E (apoE), a glycoprotein containing 299 amino acids, is associated in plasma with almost all lipoprotein particles. The majority of apoE in plasma is derived from hepatocytes, but there are some other peripheral sources among which macrophages, astrocytes and adipocytes are the most important. ApoE is mainly involved in the lipid metabolism, however so far it was revealed to be important for other processes such as neuroprotection, anti-microbial defense, oxidative stress and inflammation. Through its interaction with the LDL-receptor family members, apoE participates in the cholesterol transport [1]. In plasma, apoE mediates the clearance of lipoprotein remnants, while in the vascular wall apoE secreted by the macrophages participates in the cellular cholesterol efflux from the atheroma [2]. Macrophage-specific expression of apoE is atheroprotective even if it has no effect on the plasma lipid levels [3], [4]. Under inflammatory stress, macrophage-derived apoE is decreased [5] and hence its local beneficial effect is diminished (abolished). In the brain, the astrocytes represent the main supplier of apoE, the most abundant apolipoprotein in the cerebrospinal fluid [6]. ApoE is also expressed by adipocytes and apoE carried on lipoproteins plays an indispensable role in adipogenesis, as reviewed in [7].

**Fig. 1:**
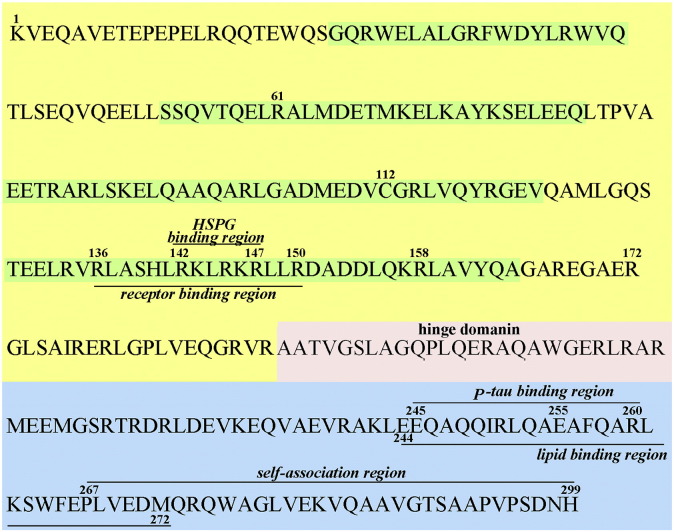
Schematic representation of apoE3 structural and functional regions. ApoE protein has two main domains joined by the hinge region (pink). The N-terminal domain (yellow) contains four α-helices (green) and includes the receptor binding region (136–150), HSPG binding region (142–147), and the amino acids 112 and 158 that vary between apoE isoforms. The C-terminal domain (blue) comprises the lipid binding domain (244–272) and apoE self-association region (267–299). Amino acids Arg^61^ and Glu^255^ are responsible for apoE domain interaction. The location of p-tau binding sequence (245–260) is also represented. (For interpretation of the references to colour in this figure legend, the reader is referred to the web version of this article.)

ApoE has two structural and functional domains [8], the N-terminal domain (amino acids 1–191) and the C-terminal domain (~ 206–299), joined by a protease-sensitive loop, as schematically represented in Fig. 1. The N-terminal domain consisting of a four antiparallel helix bundle, contains the receptor-binding region (~136–150 and Arg^172^) and the heparan sulfate proteoglycans (HSPGs) binding region, with a weak lipid-binding capability [9]. The C-terminal domain comprises amphipathic α-helices, the high-affinity lipid-binding region in the region ~244–272 and the region 267–299 responsible for apoE self-association [10]. It was determined that highly conserved regions appear to be linked to the primary apoE functions, such as ligand binding, and less conservation of amino acids was found at the ends of the protein [11].

In contrast to other mammals, humans present three isoforms of apoE, named apoE2, apoE3, and apoE4, which are the products of the ε2, ε3 and ε4 alleles [12]. The ε3 allele is the most common occurring in ~77% of the general population, while the ε2 allele accounts for ~8% and the ε4 allele for ~15%, as reviewed by [13]. The three isoforms differ in the amino acids at positions 112 and 158. ApoE3 isoform, the most prevalent isoform in normolipidemic population, has Cys^112^ and Arg^158^. Maintaining the homeostatic levels of lipids, apoE3 plays a protective role in cardiovascular diseases. ApoE2 has two cysteines (Cys^112^ and Cys^158^) and is associated with dysbetalipoproteinemia (hyperlipidemia). ApoE4 has two arginine residues (Arg^112^ and Arg^158^) and represents a risk factor for Alzheimer's disease (AD). In apoE4, Arg^61^ from the second α-helix and Glu^255^ from the C-terminal region are responsible for the “domain interaction” [14]. However, other species present Thr instead of the human Arg^61^. Interestingly, when the Arg^61^–Glu^255^ interaction is disrupted with small molecules, apoE4 binding preference shifts from VLDL to HDL particles, suggesting that the “structure correctors” converting apoE4 to an apoE3-like molecule may represent a valuable therapeutic approach [15].

In the current review, we discuss the correlation between apoE structure and its involvement in various pathologies (hyperlipidemia, cardiovascular and neurodegenerative diseases, infections and stress-related dysfunctions), in a view of prospective apoE-based therapeutic strategies.

## 2 Lipid Metabolism and Cardiovascular Disorders

The importance of apoE in lipid metabolism has been recognized decades ago. In animal models, apoE deficiency is associated with atherosclerosis [16]. Due to its high-affinity for LDL-receptor family members, apoE facilitates hepatic and extrahepatic uptake of plasma lipoproteins [1]. High density lipoproteins (HDL) represent a heterogeneous group of lipoproteins among which mature spherical particles are responsible for the atheroprotective function, promoting the removal of cholesterol from macrophages [17], [18]. Lipidation of both apoE and apoA-I leads to HDL de novo generation, but different lipids are recruited by the two apolipoproteins leading to various HDL subfractions [19]. Moreover, the binding of various proteins to HDL particles is dependent on apoE content of HDL. For instance, apoE concentration on HDL particles is important for binding of complement factor H to HDL, regulating the alternative pathway of complement cascade activation [20]. In addition, it was demonstrated that apoE genotype has high impact on apoCI concentration in plasma [21], [22]. Considering that apoE gene belongs to the cluster containing also apoCI, apoCIV and apoCII genes, and their transcription regulation is a complex process involving interactions between proximal and distal regulatory regions, a correlation between apoE and the other genes levels may be observed. Cell-specific enhancers such as HCR.1 and HCR.2 in hepatocytes [23], [24], [25], ME.1 and ME.2 in macrophages, adipocytes and astrocytes [26], [27], [28], [29] regulate the genes of the cluster through the binding of various transcription factors.

Despite the known association between apoE genotype and cardiovascular disease (CVD), in a recent large study no correlation between circulating apoE concentration and CVD events was found, suggesting that CVD risk linked to apoE genotype may be explained considering the specific functions of apoE isoforms rather than the apoE concentration in the blood [30]. A linear relationship between apoE genotypes (ε2/ε2 > ε2/ε3 > ε2/ε4 > ε3/ε3 > ε3/ε4 > ε4/ε4) and both LDL-cholesterol levels and coronary artery diseases was found [31].

Due to their structural variances, the three apoE isoforms have different receptor affinities and lipoprotein-binding preferences. Thus, apoE3 and apoE4 isoforms bind LDL-receptors, whereas apoE2 is defective [32]. However, apoE2 is not defective in binding to all LDL-receptor family members, especially LRP-1 [33]. ApoE2 and apoE3 isoforms preferentially bind to small HDL particles, while apoE4 isoform preferentially binds to large triglyceride-rich VLDL particles [34]. Nguyen et al. proposed a two-step mechanism of reversible binding of apoE to lipoproteins, involving an initial interaction and then opening of the N-terminal helix bundle domain of the apoE molecule [35]. Protein-protein interactions were found to be important for apoE binding to HDL3, while apoE-lipid interactions are important for VLDL binding [35]. ApoE lipidation status is also important for its interaction with other lipoprotein components. A recent study revealed that lipid-free apoE3 has the highest potential for binding and activation of PLTP, an important HDL modifying lipid transfer protein [36]. Interestingly, apoE lipidation abolished these differences in PLTP binding potential [36].

Transgenic mice overexpressing human apoE3 developed hypertriglyceridemia, due to stimulated VLDL triglyceride production and impaired VLDL lipolysis [37]. However, adenoviral gene transfer of human apoE isoforms in apoE-deficient mice showed that overexpression of human apoE2 increased plasma triglycerides and cholesterol; by contrast overexpression of human apoE3 or apoE4 caused neither hypertriglyceridemia nor hypercholesterolemia [38].

Plasma apoE concentration was found to be lower in apoE4 homozygotes as compared to apoE3 homozygotes [39]. Plasma lipid abnormalities associated with apoE4 can be explained by the higher rate of apoE4 catabolism as compared to apoE3. This accelerated catabolism is based on apoE4 features: a higher affinity for apoE receptor, the association with VLDL and the ability to faster convert VLDL remnants to LDL [39].

Macrophage-secreted apoE mediates cholesterol efflux, preventing cholesterol overload and the subsequent transformation into foam cells [40]. Cullen et al. demonstrated that apoE isoforms differ in the metabolism of cholesterol in human monocyte-derived macrophages in the following order: E2/2 > E3/3 ≅ E4/4 [41]. The differential role of apoE isoforms in mediating cholesterol efflux was correlated with the dissimilar secretion rates of apoE isoforms (E3/3 > E4/4 > E2/2), but also with the differences in their binding to cell surface proteoglycans and reuptake of the cholesterol-rich particles [41].

The three apoE isoforms influence intestinal absorption of cholesterol in a different manner, influencing plasma cholesterol level as following: apoE4 homozygotes had a higher efficiency of cholesterol absorption than apoE3 subjects, while the apoE2 homozygotes had the lowest values [42].

ApoE2 homozygosity often results in type III hyperlipoproteinemia (HLP), a rare inherited disorder characterized by increased levels of total cholesterol and triglycerides as well as high risk for premature atherosclerosis [43]. Interestingly, although overt hyperlipidemia requires apoE2 homozygosity [44], only a small percentage of ε2/ε2 carriers (less than 5%) develop hyperlipidemia, and the rest are either normolipidemic or even hypocholesterolemic [45]. Thus, apoE2 is necessary but not sufficient to cause HLP; other co-factors were found to predispose to the disease, such as diabetes, obesity, decreased LDL-receptor activity, hypothyroidism, low oestrogen levels, impaired lipolysis or hyperinsulinemia [46]. Besides apoE2, rare naturally occurring mutations in the apoE gene have been associated with HLP, and the majority of these mutations involve substitutions of arginine or lysine residues located within the receptor-binding region [47].

Lipoprotein glomerulopathy (LPG) is a rare kidney disorder characterized by an abnormal plasma lipoprotein profile resembling HLP, glomerular lipoprotein thrombi, proteinuria, progressive kidney failure, and increased serum apoE concentration [48]. There are many mutations in apoE gene causing LPG, but the most common mutations associated with this disorder are located in the sequence encoding LDL-receptor binding domain. Several excellent reviews covering HLP- and LPG-associated apoE mutations have already been published [47], [49].

To identify the regions of apoE responsible for generation of functional HDL particles conferring atheroprotective properties and the regions causing hypertriglyceridemia induced by systemic overexpression, a series of studies of full-length or truncated apoE expression were performed. Adenovirus-mediated gene transfer studies in mice revealed that apoE region 1–202 associated with lipoproteins efficient in the clearance of lipoprotein remnants, while region 203–299 induced hypertriglyceridemia [50]. To avoid this side effect, but still trying to correct the abnormal lipid profile, a truncated apoE lacking the C-terminal region (203–299) was generated. This truncated protein ameliorated hyperlipidemia in a human apoE2 transgenic mouse model [51]. The role of various hydrophobic residues located in region 261–283 of apoE was analyzed by Zannis and co-workers [52]. Thus, the adenoviral expression of a multiple mutant apoE4 carrying the L261A, W264A, F265A, L268A, and V269A substitutions in apoE-deficient mice was able to correct the abnormal plasma cholesterol levels without causing hypertriglyceridemia, while a mutant apoE4 bearing the W276A, L279A, V280A, and V283A substitutions failed to correct hypercholesterolemia and caused mild hypertriglyceridemia [52]. Interestingly, the first apoE4 mutant promoted the formation of spherical HDL particles, while the second apoE4 mutant, as well as the wild-type apoE4, displaced apoA-I from HDL, inducing the formation of discoidal HDL [52]. Afterwards, the group of Kypreos demonstrated that the clearance of atherogenic lipoproteins mediated by the apoE4 mutant (L261A, W264A, F265A, L268A, and V269A) was dependent on the expression of a functional LDL-receptor [53]. Interestingly, the reduction of plasma cholesterol levels in apoE-deficient mice was also obtained after bolus administration of proteoliposomes containing the recombinant mutant (L261A, W264A, F265A, L268A, and V269A) apoE4 protein [53]. Further studies showed that the triple substitution with alanine of Leu^261^, Trp^264^, and Phe^265^ residues in apoE2 or apoE4 promoted the formation of spherical HDL particles and prevented the hypertriglyceridemia in apoE- and apoAI-deficient mice [54]. The study of Georgiadou et al. determined the structural and thermodynamic properties of the above mentioned apoE mutants and revealed the potential use of mutated apoE forms for therapeutic applications in order to correct the remnant removal disorders [55].

Other studies demonstrated that the domain 1–185 of human apoE is sufficient for the formation of apoE-containing HDL particles in apoA-I deficient mice, but the longer truncated variants are more efficient in both raising HDL levels and decreasing the triglyceride-rich lipoproteins concentration [56]. Using apoE4 truncated variants, Vezeridis et al. found that apoE4-185 and apoE4-202 generated only discoidal HDL particles, while apoE4-229, apoE4-259 and full-length apoE4 generated discoidal and spherical HDL particles [57]. In addition, they found that all apoE4 truncated forms were able to promote ABCA1-dependent cholesterol efflux *in vitro*, although less efficiently that full-length apoE4. Using truncated forms of apoE4, Dafnis et al. determined the domain required for PLTP binding and activation to be located within the amino terminal 1–185 region [36].

To exploit the beneficial properties of apoE, efforts to create synthetic peptides containing certain regions of apoE were made. The effects of a dual-domain apoE peptide (Ac-hE18A-NH2, the fragment 141–150 containing the putative receptor-binding region of human apoE, covalently linked to a class A amphipathic helix, 18A) on the reduction of plasma cholesterol in mice and rabbits as well as their potential use as a drug in humans have been previously reviewed [58]. Another apoE peptide (EpK) containing the 141–150 N-terminal region, a six-lysine linker and the region 234–254, was generated [59], and it was found that it preferentially bound to HDL, improving its functions regarding the cholesterol efflux and the inhibition of LPS-induced pro-inflammatory cytokine expression in macrophages. Recently, Xu et al. produced another human apoE peptide (hEp), containing almost the entire helix four of the N-terminal region and the major C-terminal lipid-binding region of apoE, which improved its receptor-binding and lipid-binding abilities [60]. They reported that lentivirus-mediated hEp expression reduced the lipid accumulation and the formation of atherosclerotic lesions in aged apoE-deficient mice [60]. Thus, these findings encourage the therapeutic use of recombinant apoE proteins to correct cardio-metabolic disorders.

## 3 Neurodegenerative Disorders

Due to its function in lipid transport, apoE plays a significant role in the brain homeostasis, regulating the lipid and glucose metabolism, neuronal signaling, being effective in neuronal cells preservation and remodeling [61], [62], [63]. In the brain, the astrocytes provide apoE which is assembled in lipoproteins and then transported to neurons where is taken up via LDL-receptor superfamily members localized on the surface of the neurons [64].

Neuropathology studies revealed that apoE4 isoform is a risk factor for Alzheimer's disease (AD), a gradual neurodegenerative disorder that includes cognitive decline leading to dementia [65]. Wishart et al. determined the differences in brain activation during working memory in ε4/ε3 healthy individuals as compared to ε3/ε3 carriers [66]. Information obtained by positron emission tomography may represent a valuable method for early diagnosis of asymptomatic AD patients. The main features of AD are the presence of the amyloid plaque in the extracellular space and of the intracellular neurofibrillary tangles, as a consequence of *tau* protein hyperphosphorylation [67]. The reasons of apoE4 association with AD have not been elucidated. However, data from the literature highlight some possible mechanisms though which apoE4 is involved in AD initiation and progression. Thus, it was revealed that apoE4 has a lower affinity for amyloid β than apoE3 [68]. Consequently, an impaired clearance of amyloid β accelerates its aggregation and accumulation. The affinity of apoE isoforms for amyloid β is affected by the lipidation status [69]. The features of the interaction between apoE and amyloid β are reviewed in [70]. Huang et al. showed that apoE4 fragments similar to those found in the brains of AD patients affected various AD-related pathological processes [67]. Because of its conformation and reactivity, apoE4 is more susceptible to proteolysis than apoE3 [67], and the cleavage products are injurious to neuronal repair, cause neurotoxicity [65] and promote AD-like neurodegeneration [71].

Numerous epidemiological studies revealed that ε4 female carriers present a higher risk of AD (approx. twofold higher) as compared to ε4 male carriers, as reviewed in [72]. The role of sex differences in apoE4 effects on AD was confirmed in animal models. In a quadruple transgenic mice model for AD (mice bearing apoE4 as well as transgenes for APPSwe, PS1M146V and tauP301L) significant memory impairment and higher levels of amyloid β species, β-site APP cleavage enzyme (BACE-1) and one BACE-1 inducer -transcription factor Sp1 were revealed in the hippocampal tissue of female mice as compared to their male counterparts [73].

Recently, Lane-Donovan and Herz revealed that diet influences hippocampal apoE protein levels in an isoform-dependent manner suggesting that apoE genotype and dietary intervention play role in the prevention strategy for AD [74]. Hippocampal apoE levels were reduced by a high-fat diet when mice expressed human apoE3 isoform, but not when mice expressed murine apoE or apoE4 isoform or when the mice received a ketogenic diet (high-fat, low-carbohydrate); in contrast, a high-fat diet increased plasma apoE levels in all genotypes, while a ketogenic diet augmented plasma apoE levels only in apoE4 transgenic mice [74].

Although the presence of apoE2 isoform is protective in the context of AD, apoE2 was suggested as a biomarker of susceptibility for posttraumatic stress disorder (PTSD) since its presence increased the incidence and the severity of this debilitating mental disorder in veterans carrying apoE2 as well as in mice expressing human apoE2 [75]. Using transgenic mice expressing human apoE4, apoE3 or apoE2 in the presence or in the absence of LDLR, Johnson et al. showed that anxiety-like behavior and cued memory are influenced by apoE isoforms (E4 > E3 > E2) and suggested that these processes occur via an LDL-receptor independent mechanism [76].

Numerous studies using truncated forms of apoE4 were performed in order to identify the region responsible for the detrimental effects of apoE4 that contribute to AD pathology. Studies on human neuroblastoma cells revealed that the apoE4-165 fragment, in which residues 166–299 are removed, had deleterious effects on amyloid β clearance and reactive oxygen species production [77]. Further work of the same group demonstrated that longer or shorter deletion fragments did not exhibit the same effects [78]. This finding was explained by the folding of the apoE4–165 fragment that presents a more helical structure, as determined by circular dichroism measurements and thermodynamic analysis. The compact structure and thermodynamic properties of region 1–165 of apoE4 represent distinctive features involved in neurodegeneration. A phenotype similar to that observed for apoE4-165 was determined for the natural apoE4-L28P mutant, highlighting the important role of apoE4 in intraneuronal accumulation of Aβ [79]. This study also suggests that the structural integrity of apoE4 is important for its role in AD pathogenesis.

To determine the region of apoE responsible for the interaction with phosphorylated *tau* protein, the group of Mahley [67] performed transfection experiments on Neuro-2a cells with C-terminal truncated forms (1–271) of apoE3 and apoE4. The results showed that both apoE3 and apoE4 truncated forms interact with phosphorylated *tau* proteins, but apoE4 is more potent in inducing the cytoskeletal alteration and formation of the intracellular neurofibrillary tangles inclusions [67]. Furthermore, the apoE region which interacts with phosphorylated *tau* protein and forms the intracellular neurofibrillary tangles inclusions was identified. Transfection experiments in Neuro-2a cells using N-terminal truncated apoE showed that residues 245–260 are responsible for this feature [67].

Dafnis et al. revealed the involvement of apoE4-truncated forms in neuroinflammation [80]. Their results showed that in SK-N-SH neuroblastoma cells, the fragment 1–185 of apoE4 regulates the levels of matrix metalloproteinase 9 (MMP9) and tissue inhibitor of metalloproteinase 1 (TIMP1) through the modulation of the level of inflammatory molecules: increases IL-1β and decreases IL-10 expression [80]. Modifications in MMP9 level which contribute to Aβ clearance or TIMP1 expression, can be correlated with AD pathogenesis [81]. In human astrocytoma SW-1783 cells, both full-length and truncated form of apoE4 (1–185) contributed to TIMP1 enhancement, without any effect on MMP9, through a suggested mechanism involving a decrease of TNFα expression [80]. Thus, the fragment 1–185 of apoE4 increased the levels of TIMP1 in the two cell lines (SK-N-SH and SW-1783) by a different mechanism. Overexpression of apoE4 fragment 1–272 induced its binding to various components of mitochondrial complexes and promoted mitochondrial dysfunction in Neuro-2a cells [82]. On the other hand, apoE 133–149 peptide displayed neuroprotective functions, besides its anti-inflammatory properties [83].

From studies using a humanized apoE transgenic mouse model (Arg^61^ apoE mice), it has been concluded that due to the “domain interaction”, apoE4 is recognized as misfolded, accumulates and activates the endoplasmic reticulum stress response, thus inducing astrocyte dysfunction [84]. Noteworthy, the mutation of Arg^61^ to Thr in apoE4, as well as the “structure correctors” which abolish the domain interaction, rescued apoE impaired intracellular trafficking through the endoplasmic reticulum and the Golgi apparatus and decreased its storage in the endoplasmic reticulum [85], [86]. GIND25 (Azocarmine G), a disulfonate that abolishes apoE4 domain interaction decreased amyloid β production induced by apoE4 to levels very similar to those induced by apoE3 [65]. PH002, a newly identified phthalazinone derivative was found to be a more potent disruptor of apoE4 domain interaction than GIND25 [86]. As it has been already stated, the “structure correctors” could be used to convert apoE4 into an apoE3-like molecule, representing promising candidates for the treatment of apoE4-related disorders [87].

## 4 Infectious Diseases

Many studies have shown that apoE is also implicated in viral infections. ApoE4 isoform has been linked to certain infections caused by herpes simplex virus type-1 (HSV-1), hepatitis C virus (HCV) and human immunodeficiency virus (HIV) [88].

HSV-1 was found frequently in the brain of elderly normal subjects and AD patients [89], [90]. It was suggested that apoE4 isoform can be responsible for a facilitated entry of HSV-1 particles into the cell [88]. Moreover, it was demonstrated that apoE4 is able to promote the viral colonization of the brain with higher efficiency than apoE3 [91]. However, a variety of genes and proteins important for AD development are affected by the HSV-1 infection, as reviewed by Piacentini et al. [92].

HCV needs apoE for its assembly/infection, and the host lipid metabolism is involved in the viral infection, as reviewed in [93]. All the three apoE isoforms modulated the assembly and infectivity of HCV in a similar manner in cell cultures [94]. Surprisingly, it was reported that apoE4 confers protection for HCV infection [95]. Mutagenesis studies showed that production of HCV required the interaction between apoE and nonstructural protein 5A (NS5A), which plays a role in HCV replication, through its C-terminal domain [94], [96]. Studies using progressive deletions revealed that amino acids 201–299 of apoE interact with NS5A, while the apoE fragment containing only residues 211–299 failed to interact with NS5A. It was found that the NS5A binding domain on apoE is located between amino acids 205 and 280. Consequently, when this region was removed, apoE-NS5A interaction was disrupted, and HCV assembly failed. Even if the N-terminal domain seems to be unnecessary for the interaction of apoE with NS5A, this domain is required for the enhancement of apoE activity in HCV production because it contains the receptor binding region that mediates HCV infection [93].

HIV infection was correlated with apoE isoforms. It was demonstrated that apoE4 increased the rate of HIV cell entry as well as the disease progression, as determined by the study of a large cohort of HIV-positive subjects [97]. The authors speculated that the differences in the cholesterol binding affinities of the apoE isoforms results in the contrasting behavior of apoE4 and apoE3 related to the HIV attachment and fusion, since cholesterol is a main component of the viral envelope [97]. The amphipathic helix domain of apoE3 binds to gp41 protein and inhibits HIV infection, in a similar manner with T-20, a clinical inhibitor [98].

Besides the multiple functions of apoE, it has been stated that apoE also has immunomodulatory functions and protective role in Gram-negative sepsis [99].

Considering the role of apoE in various infections, antimicrobial peptides derived from apoE with therapeutic potential were synthesized. ApoE 133–150 peptide was found to be lethal for several Gram-positive (*Staphylococcus aureus*, *Bacillus subtilis*) and Gram-negative (*Pseudomonas aeruginosa*, *Escherichia coli*, *Klebsiella pneumoniae*) bacteria, but displayed no toxicity on several human cell cultures [83]. Furthermore, this peptide reduced the expression of pro-inflammatory cytokines in LPS-treated THP-1 macrophages. A longer apoE peptide (133–162) possessed an antimicrobial activity against *E. coli*, *P. aeruginosa*, *S. aureus*, and *Salmonella* similar to Gentamicin and LL-37, a neutrophil-derived antibiotic peptide [100]. It was shown that apoEdp, a tandem repeated peptide 141–149, had antimicrobial effects on viruses (HSV1, HIV and HCV), bacteria (*S. aureus*) [88] and parasites (*Plasmodium* spp) [101]. Infection of hepatocytes Hepa 1–6 with *Plasmodium berghei* indicated that apoE 141–149 inactivated the sporozoites by lysis, while substitution Leu → Trp in the tandem peptide blocked the microorganisms adherence by decreasing HSPGs availability [101]. Another synthetic peptide, apoE23, generated by joining sequences 141–148 and 135–149, exhibited a higher microbiological activity than apoEdp. ApoE23 includes a linker of five amino acids (RLASH) between the repeated 141–148 apoE domains [83].

Taken together, these data demonstrate that apoE peptides possess antimicrobial activities without adverse effects and may have therapeutic potential against pathogenic microorganisms.

## 5 Stress-related Pathological States

Data showed that apoE prevents lipid oxidation, a property based, at least in part, on apoE capacity to bind metal cations [102]. The region of apoE conferring protection against LDL oxidation was identified to be located between the residues 141–155, which overlaps with the receptor-binding domain [103]. The anti-oxidative properties of apoE are isoform-dependent in the following order: apoE2 > apoE3 > apoE4 [104]. Studies on post-mortem brains of AD patients showed that apoE4 is correlated with elevated lipid peroxidation and hydroxyl radical levels in blood [104].

The levels of anti-oxidative and anti-inflammatory metallothioneins [105], as well as of serum paraoxonase [106] were lower in apoE4, as compared to apoE3 transgenic mice. Moreover, the expression of Nrf2 and its target genes such as glutathione-S-transferase, NAD(P)H dehydrogenase and heme oxygenase-1 were lower in apoE4 mice than in apoE3 mice [107].

*In vitro* studies showed that macrophages overexpressing apoE4, stimulated with LPS and PMA, displayed significant membrane oxidation and generated higher nitric oxide and superoxide anion radicals, as compared with stimulated apoE3-secreting macrophages [108]. Interestingly, to counterbalance the oxidative stress, heme oxygenase-1, an anti-inflammatory protein, was increased as a stress-response in apoE4-expressing macrophages treated with LPS [109]. ApoE exerts local anti-inflammatory effects by promoting the conversion of macrophages from the proinflammatory M1 to the anti-inflammatory M2 phenotype, as it has been already stated in [110]. Moreover, apoE is able to reduce the production of IL-2, by suppression of CD4+ and CD8+ lymphocytes [111].

The differential association of the apoE isoforms with mitochondrial dysfunction and endoplasmic reticulum stress response was recently reviewed [112]. It was suggested that in neurons apoE4 is prone to protease cleavage generating apoE4 fragment 1–272 that binds to several components of mitochondrial complexes and subsequently initiates mitochondrial dysfunction [82].

## 6 Conclusions

The structural differences between the apoE isoforms translate into significant functional changes with implications in pathophysiological conditions including dyslipidemia, cardiovascular diseases, neurodegenerative disorders, infections and inflammatory states.

Regarding apoE involvement in lipid metabolism, site-specific mutated or truncated apoE gave valuable clues on various regions and residues of apoE responsible for hypertriglyceridemia and athero-genesis/protection. Based on isoform-dependent affinities for the receptors, lipids and proteins, it was revealed that the apoE isoforms are associated with various lipoproteins and differentially contribute to cholesterol efflux.

ApoE plays a pivotal role in maintaining the neuronal function, providing the brain with the necessary cholesterol. However, apoE4 is considered a risk factor for AD. The complete mechanism of apoE4 involvement in AD initiation and/or progression is not elucidated, despite that a lower affinity of apoE4 for amyloid β was revealed, leading to a less efficient clearance of amyloid β; in addition, apoE4 proteolysis generates cleavage products that may aggravate the disease.

ApoE intersection with various viral intracellular pathways influences the disease evolution. The apoE isoforms were differently correlated with viral infections, influencing the viral cell entry and infection progression.

In stress-related pathological states and inflammation, apoE plays important roles such as: prevention of lipid oxidation, and macrophage polarization. The anti-oxidative properties of apoE are also isoform-dependent.

Noteworthy, the beneficial properties of apoE were exploited using mimetic apoE peptides. Recombinant or synthetic peptides derived from apoE possess pharmacological potential, lowering plasma cholesterol, exerting antimicrobial activity and immunomodulatory effects. Some of these peptides were already introduced in clinical trials and presented promising results.

Despite the intensive research and the innovative approaches that targeted this intriguing protein, there is yet no strategy that could be entirely recommended for apoE-related disorders, but there are several promising candidates, such as apoE mimetic peptides or specific structure correctors that could be used in personalized medicine.

## Conflict of Interest

The authors declare no conflict of interest.
